# Paediatric mpox during Sierra Leone’s 2024 to 2025 clade IIb outbreak: A national surveillance and outbreak-investigation study of 415 children

**DOI:** 10.1371/journal.pntd.0014785

**Published:** 2026-10-07

**Authors:** Eric Nzirakaindi Ikoona, Lucy Namulemo, Mary Magdalene Sinnah, Mohamed Alex Vandi, Foday Sahr

**Affiliations:** 1 National Public Health Agency (NPHA), Freetown, Sierra Leone; 2 School of Professional Counseling, Lindsey Wilson University, Columbia, Kentucky, United States of America; The Pennsylvania State University, UNITED STATES OF AMERICA

## Abstract

**Background:**

Evidence on paediatric mpox during contemporary African clade IIb outbreaks remains limited. We described paediatric cases in Sierra Leone’s 2024–2025 clade IIb G.1 outbreak by person, time, and place and examined clinical care, outcomes, vaccination records, and documented exposures.

**Methodology/Principal findings:**

We conducted a retrospective descriptive study using the national District Health Information Software 2 (DHIS2) mpox database, supplemented by patient charts and outbreak-investigation forms. Among 5,466 cases with recorded age, 415 (7.6%) were children younger than 15 years; 188 (45.3%) were aged 0–4 years, 121 (29.2%) were aged 5–9 years, and 106 (25.5%) were aged 10–14 years. Of 415 paediatric cases, 411 (99.0%) were laboratory-confirmed. Outcome was recorded for 261 children (62.9%); three died (1.1%, exact 95% CI 0.2 to 3.3). Care location was recorded for 285 children; 216 (75.8%) received hospital care for isolation, clinical management, or both. Three sporadic paediatric cases of mpox-like illness were reported from 2017 through 2024; notifications then rose rapidly from April 2025 and peaked in May. Western Area Urban accounted for 195/413 cases with known district (47.2%); crude notified paediatric rates were highest in Western Area Urban, Koinadugu, and Falaba. Household or caregiving contact was the most common documented exposure among 246 classifiable investigation forms (141, 57.3%). Mpox vaccination status was recorded for 27/54 cases aged 12–14 years; six were vaccinated.

**Conclusions/Significance:**

Children were not spared during Sierra Leone’s clade IIb outbreak. Response plans should include children in case finding, contact follow-up, clinical care, and vaccination assessment. DHIS2 should capture epidemiological links, clinical outcomes, vaccine eligibility and status, and potential exposure settings through standardised fields.

## Author summary

The 2022 global mpox outbreak focused attention on adults, who comprised most reported cases. We analysed Sierra Leone’s national surveillance database, patient charts, and outbreak-investigation forms to describe paediatric mpox during the country’s 2024–2025 clade IIb outbreak. Children accounted for 415 of 5,466 cases with recorded age, and almost half of paediatric cases occurred in children younger than 5 years. Three children died among those with recorded outcomes. National records documented three sporadic paediatric cases of mpox-like illness from 2017 through 2024. Notifications then rose rapidly from April 2025 and peaked in May. Western Area Urban recorded the most paediatric cases, while crude notified rates were also high in Koinadugu and Falaba. Household or caregiving contact was the most common documented exposure in available investigation forms, but the records could not establish how individual children acquired infection. Mpox response should identify child contacts early, investigate local exposure patterns, and record linked cases, clinical outcomes, and vaccine eligibility consistently. Prospective contact studies can clarify transmission pathways and guide age-appropriate prevention.

## Introduction

Mpox is caused by monkeypox virus. Clade I circulates mainly in Central Africa, whereas clade II includes the clade IIb viruses associated with sustained human transmission since 2022 [1–3]. Children carried a substantial burden in earlier African outbreaks, but children and adolescents comprised only 1.3% of reported cases in the 2022 multicountry outbreak [4,5]. Subsequent African reports show marked variation in paediatric involvement: children represented a large share of confirmed cases across African Union member states in 2024, and those younger than 5 years had the highest attack rate in a Ugandan study [6–12].

National Public Health Agency (NPHA) surveillance records documented three sporadic paediatric cases of mpox-like illness from 2017 through 2024. Sierra Leone then experienced rapid national expansion of a clade IIb G.1 outbreak in 2025, and genomic analysis indicated sustained local transmission before public detection [13–15]. National District Health Information Software 2 (DHIS2) records, patient charts, and outbreak-investigation forms provided complementary epidemiological, clinical, and exposure data.

We described mpox among children younger than 15 years by person, time, and place and summarised clinical features, care, outcomes, modified vaccinia Ankara-Bavarian Nordic (MVA-BN) vaccination records, documented exposures, and data completeness. Cases aged 15 years or older provided context for selected geographic and surveillance measures.

## Methods

### Ethics statement

The Sierra Leone Ethics and Scientific Review Committee approved this secondary analysis of de-identified surveillance, chart, and outbreak-investigation data (SLESRC-2025–049) and confirmed that individual informed consent was not required because the records were collected routinely and no participant contact occurred. No direct identifiers are reported. Fatal-case descriptions use broad age categories and omit exact dates and locations; these generalisations do not alter the clinical interpretation. The analysis was conducted under the NPHA public health mandate.

### Study design, setting, and cohort

We conducted a retrospective national descriptive study of paediatric mpox during Sierra Leone’s 2024–2025 outbreak. We described cases by person, time, and place using the NPHADHIS2 mpox database and supplemented these data with available patient charts and original outbreak-investigation forms. We reviewed NPHA surveillance summaries from 2017 through 2024 for temporal context; their aggregate count was not added to the 415-case analytic cohort. Sierra Leone declared a public health emergency in January 2025; the Ministry of Health announced the end of the outbreak in December 2025 [16]. We analysed a database extract frozen on 1 July 2026. DHIS2 forms part of the national integrated disease surveillance and response system [17].

Eligible records received a final national classification of laboratory-confirmed, probable, or clinician-diagnosed. The public outbreak-end report listed 5,442 confirmed cases [16], whereas the later frozen extract contained 5,445 laboratory-confirmed, 20 probable, and 3 clinician-diagnosed records (5,468 total). These totals represent different data snapshots; we excluded no eligible records. Age was missing for two records, leaving 5,466 age-classifiable cases: 415 children younger than 15 years and 5,051 people aged 15 years or older (S1 Fig). We grouped children as 0–4, 5–9, and 10–14 years. We used final case classification because the separate specimen-result field was incomplete and did not capture repeat or second-specimen testing.

### Variables and supplementary record review

DHIS2 variables included age, sex, case classification, symptoms, lesion sites, complications, care location, MVA-BN vaccination, HIV status, symptom-onset date, first facility-contact date, district of residence, and final outcome. We defined time as epidemiological week of symptom onset, using first facility-contact date when onset was missing, and place as district of residence. Mortality was the proportion of deaths among children with a recorded final outcome. A marked binary clinical field indicated documented presence; an unmarked field was coded as not documented because DHIS2 did not distinguish absence from non-entry. The field labelled ‘vaginal lesions’ did not distinguish vulval, perineal, or intravaginal location. DHIS2 lacked complete national fields for lesion count, mucosal or ocular involvement, nutritional status, treatment, exposure route, household links, child-protection referral, and recovery time.

We sought patient charts for all children recorded as receiving hospital care and for all children with a recorded death. We abstracted the purpose of admission (isolation, clinical management, or both), lesion burden and distribution, mucosal or ocular involvement, hydration and nutritional status, comorbidities, treatment, complications, admission and discharge dates, outcome, and the recorded cause or circumstances of death.

For each paediatric case, we sought the original mpox outbreak-investigation form completed by district response teams during routine case and contact investigation. We abstracted household links; caregiving; shared sleeping space, bedding, clothing, or other materials; school, peer, community, and health-care contacts; animal, wildlife, or bushmeat exposure; follow-up; and referrals. For children with genital or perianal lesions, we also recorded any assessment of possible sexual contact or abuse and any social-work, child-protection, forensic, or medico-legal review. DHIS2 supplied national denominators; chart and form analyses included retrieved records with information for the relevant variable.

### Exposure and vaccination definitions

We assigned a primary exposure category when the form identified one predominant exposure. Household or caregiving exposure included a household case, direct care, shared sleeping space, shared bedding or clothing, bathing, feeding, or similar domestic contact. School, peer, or community exposure required documented non-household close contact. Animal-related exposure included contact with rodents or other wild animals, hunting, or bushmeat handling or preparation. Forms with several plausible exposures but no predominant source were classified as ‘no single exposure identified’; forms with insufficient information were unclassifiable. These categories describe documented exposure histories, not routes of infection.

MVA-BN was the vaccine used during the response. We ascertained vaccination status from the DHIS2 field and patient charts where available. The World Health Organization (WHO) recommends mpox vaccination during outbreaks and prequalified MVA-BN for adolescents aged 12–17 years [18,19]. During limited supply, Sierra Leone used a targeted single-dose campaign for selected groups aged 12 years or older, including contacts and other high-risk groups [20]. We therefore restricted the paediatric vaccination analysis to children aged 12–14 years with recorded MVA-BN status. We excluded younger entries because the data could not distinguish MVA-BN from routine childhood immunisation, exceptional use, or miscoding. Vaccination date, dose, indication, eligibility, and timing relative to exposure or onset were inconsistently documented; we summarised recorded status and did not estimate coverage, access, or effectiveness.

### Statistical analysis

We summarised categorical variables as n/N (%) using field-specific denominators and continuous variables as medians with interquartile ranges (IQR). The prespecified objective was descriptive, and no aetiological hypothesis was specified. The mortality proportion includes an exact binomial 95% confidence interval (CI). We plotted laboratory-confirmed paediatric cases by epidemiological week. District counts and crude notified rates used provisional 2021 populations [21] and a projected 2025 national population share of 40.1% younger than 15 years [22]; the remaining 59.9% represented people aged 15 years or older. Because district testing, care-seeking, case ascertainment, and age structure may differ, these rates measure notified burden rather than incidence. Statistical analyses used Python 3.12. The district map was created in R version 4.6.1 (R Foundation for Statistical Computing, Vienna, Austria) using Sierra Leone administrative boundary shapefiles produced by the Government of Sierra Leone and distributed through the Humanitarian Data Exchange (https://data.humdata.org/dataset/cod-ab-sle) under the Creative Commons Attribution 3.0 IGO licence.

## Results

### Person

The extract contained 5,468 eligible cases, of which 5,466 had age recorded; 415/5,466 (7.6%) were children younger than 15 years (S1 Fig).

Median paediatric age was 5 years (IQR 1–10). Of 415 children, 188 (45.3%) were aged 0–4 years, 121 (29.2%) were aged 5–9 years, and 106 (25.5%) were aged 10–14 years; 230 (55.4%) were male (Table 1). Final classification was laboratory-confirmed in 411 children, probable in four, and clinician-diagnosed in none. In the full national cohort, 5,445/5,468 cases were laboratory-confirmed.

**Table 1:** Selected demographic and clinical characteristics of paediatric mpox cases by age group.

Characteristic	0 to 4 years (n = 188)	5 to 9 years (n = 121)	10 to 14 years (n = 106)	All children (n = 415)
Final classification: laboratory-confirmed	187/188 (99.5)	120/121 (99.2)	104/106 (98.1)	411/415 (99.0)
Male sex	109/188 (58.0)	65/121 (53.7)	56/106 (52.8)	230/415 (55.4)
Fever documented	161/188 (85.6)	99/121 (81.8)	86/106 (81.1)	346/415 (83.4)
Rash documented	156/188 (83.0)	103/121 (85.1)	87/106 (82.1)	346/415 (83.4)
Arm lesions documented	129/188 (68.6)	87/121 (71.9)	71/106 (67.0)	287/415 (69.2)
Leg lesions documented	126/188 (67.0)	82/121 (67.8)	76/106 (71.7)	284/415 (68.4)
Face lesions documented	114/188 (60.6)	74/121 (61.2)	56/106 (52.8)	244/415 (58.8)
Secondary bacterial skin infection documented	13/188 (6.9)	10/121 (8.3)	13/106 (12.3)	36/415 (8.7)
Hospital care (recorded care location)	103/136 (75.7)	60/83 (72.3)	53/66 (80.3)	216/285 (75.8)
HIV negative (recorded HIV status)	39/39 (100.0)	16/16 (100.0)	21/21 (100.0)	76/76 (100.0)
DHIS2 ‘vaginal-lesion’ field (girls)	13/79 (16.5)	8/56 (14.3)	22/50 (44.0)	43/185 (23.2)
Perianal lesions documented	8/188 (4.3)	4/121 (3.3)	5/106 (4.7)	17/415 (4.1)
Deaths among recorded outcomes	2/123 (1.6)	1/76 (1.3)	0/62 (0.0)	3/261 (1.1)

Note: Values are n/N (%) unless stated; denominators vary by field completeness. Probable classifications numbered 1, 1, and 2 in children aged 0–4, 5–9, and 10–14 years, respectively; no paediatric case was clinician-diagnosed. For binary DHIS2 checkbox fields, numerators indicate documented presence, and unmarked fields were treated as not documented. The field labelled ‘vaginal lesions’ did not define exact anatomy; estimates are restricted to girls, and three male records with this checkbox were treated as field errors. MVA-BN status is reported separately for eligible children aged 12–14 years.

Fever and rash were each documented in 346/415 children (83.4%); arm and leg lesions were the most common sites. Secondary bacterial skin infection was documented in 36/415 children (8.7%), and acute respiratory distress syndrome in one. Care location was known for 285 children: 216 received hospital care and 69 home care.

Final outcome was recorded for 261 children; three died (1.1%, exact 95% CI 0.2 to 3.3). Their onset-to-care intervals were 6, 15, and 27 days. Two records described poor feeding or dehydration, one secondary bacterial skin infection, and one moderate acute malnutrition. HIV status was unavailable for all three (Table 2).

**Table 2:** Clinical summary of the three paediatric deaths.

Field	Case 1	Case 2	Case 3
Confirmation	Laboratory-confirmed	Laboratory-confirmed	Laboratory-confirmed
Age group and sex	1 to 4 years, female	5 to 9 years, male	Younger than 1 year, female
Onset-to-care interval	15 days	6 days	27 days
Admission context	Clinical management and isolation after delayed presentation	Clinical management and infection control	Facility care after prolonged delay before first recorded contact
Clinical findings	Generalised rash, fever, poor feeding, and dehydration recorded	Fever, generalised rash, and secondary bacterial skin infection recorded	Diffuse rash, poor feeding, and dehydration recorded
HIV, nutrition, and comorbidities	HIV status not recorded; nutrition assessment not recorded; no chronic comorbidity documented	HIV status not recorded; nutrition assessment not recorded; no chronic comorbidity documented	HIV status not recorded; moderate acute malnutrition documented
Treatment and complications	Oral or intravenous fluids, antipyretic treatment, and skin care recorded; clinical impression included severe mpox with dehydration	Antibiotic treatment for suspected secondary bacterial infection recorded; clinical impression included severe mpox with suspected sepsis	Fluids and supportive care recorded; death occurred shortly after admission

Note: Table 2 draws on DHIS2, patient charts, and outbreak-investigation forms. Exact dates, ages, and locations were generalised to reduce re-identification risk; onset-to-care intervals were retained. These generalisations do not alter the clinical interpretation. The summaries provide clinical context but do not support analysis of mortality predictors because documentation of HIV status, nutrition, complications, treatment, and cause of death was not standardised.

Among 54 children aged 12–14 years, MVA-BN status was recorded for 27, of whom six were vaccinated. Vaccination date, dose, indication, eligibility, and timing relative to exposure or symptom onset were inconsistently documented. HIV status was recorded for 76/415 children; all were recorded as HIV negative.

The DHIS2 ‘vaginal lesions’ field was marked for 43/185 girls: 13/79 aged 0–4 years, 8/56 aged 5–9 years, and 22/50 aged 10–14 years. Perianal lesions were documented in 17/415 children. Three male records with the ‘vaginal lesions’ box marked were treated as nonspecific field errors.

### Time

NPHA surveillance summaries documented three sporadic paediatric cases of mpox-like illness from 2017 through 2024. The epidemic curve included 411 laboratory-confirmed paediatric cases from the outbreak cohort. Notifications remained low through early 2025, rose sharply in April, and peaked at 37 in the week ending 18 May 2025. Counts declined after June, with a smaller increase in September and early October (Fig 1).

**Fig 1:**
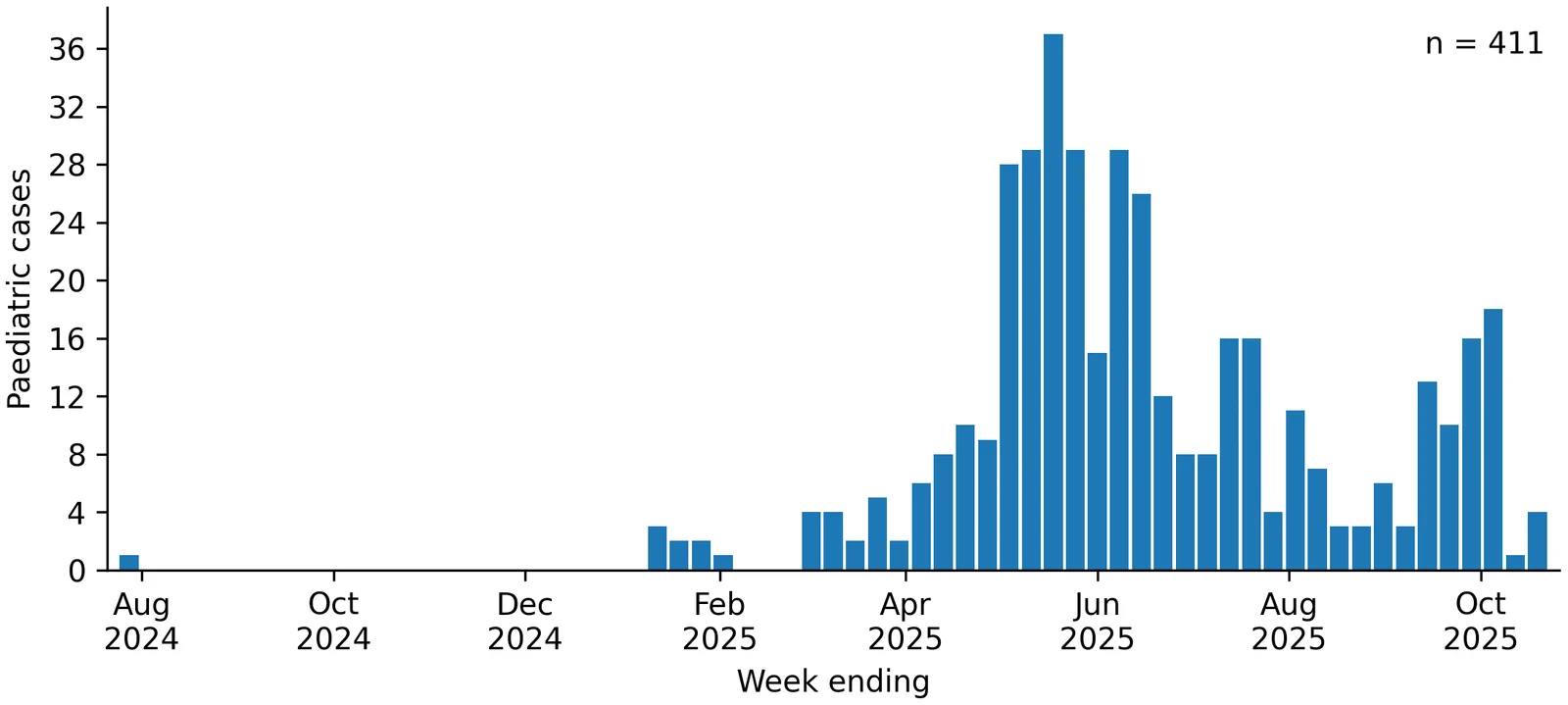
Weekly epidemic curve of laboratory-confirmed paediatric mpox cases, Sierra Leone, 2024 to 2025. Bars show cases by epidemiological week of symptom onset; first facility-contact date was used when onset was unavailable.

### Place

District was known for 413 children. Western Area Urban recorded 195/413 paediatric cases (47.2%). Koinadugu, Falaba, and Karene together recorded 83/413 (20.1%), compared with 79/5,041 cases aged 15 years or older with known district (1.6%).

Crude notified paediatric rates were highest in Western Area Urban (80.2 per 100,000 estimated children), Koinadugu (60.5), and Falaba (30.0). The estimates used provisional district populations and a uniform national age projection and represent notified burden rather than incidence (Fig 2; Table 3).

**Table 3:** District distribution and crude notified rates among paediatric cases and cases aged 15 years or older.

District	Paediatric cases	Paediatric rate/100,000 children	Cases aged 15 years or older	Rate/100,000 people aged 15 years or older
Western Area Urban	195	80.2	3,141	864.3
Koinadugu	50	60.5	54	43.7
Falaba	20	30.0	10	10.0
Bombali	25	16.1	304	131.1
Western Area Rural	40	15.1	726	183.1
Port Loko	26	12.3	183	57.9
Karene	13	11.2	15	8.6
Tonkolili	11	4.9	74	22.2
Kambia	5	3.4	59	26.8
Kenema	10	3.2	126	27.2
Kono	8	3.2	125	33.6
Moyamba	2	1.4	26	12.5
Kailahun	3	1.4	22	6.7
Bo	3	1.0	112	24.7
Bonthe	1	0.8	45	25.2
Pujehun	1	0.6	19	7.4

Note: Districts are ordered by paediatric notification rate. Rates use provisional 2021 district populations [21]. We estimated the population younger than 15 years by applying the projected national 2025 share of 40.1% to each district [22] because district-specific age structures were unavailable. Rates represent notified burden, not incidence, and may be overestimated where children comprise more than 40.1% of the district population. District was missing for two paediatric cases and 10 cases aged 15 years or older.

**Fig 2:**
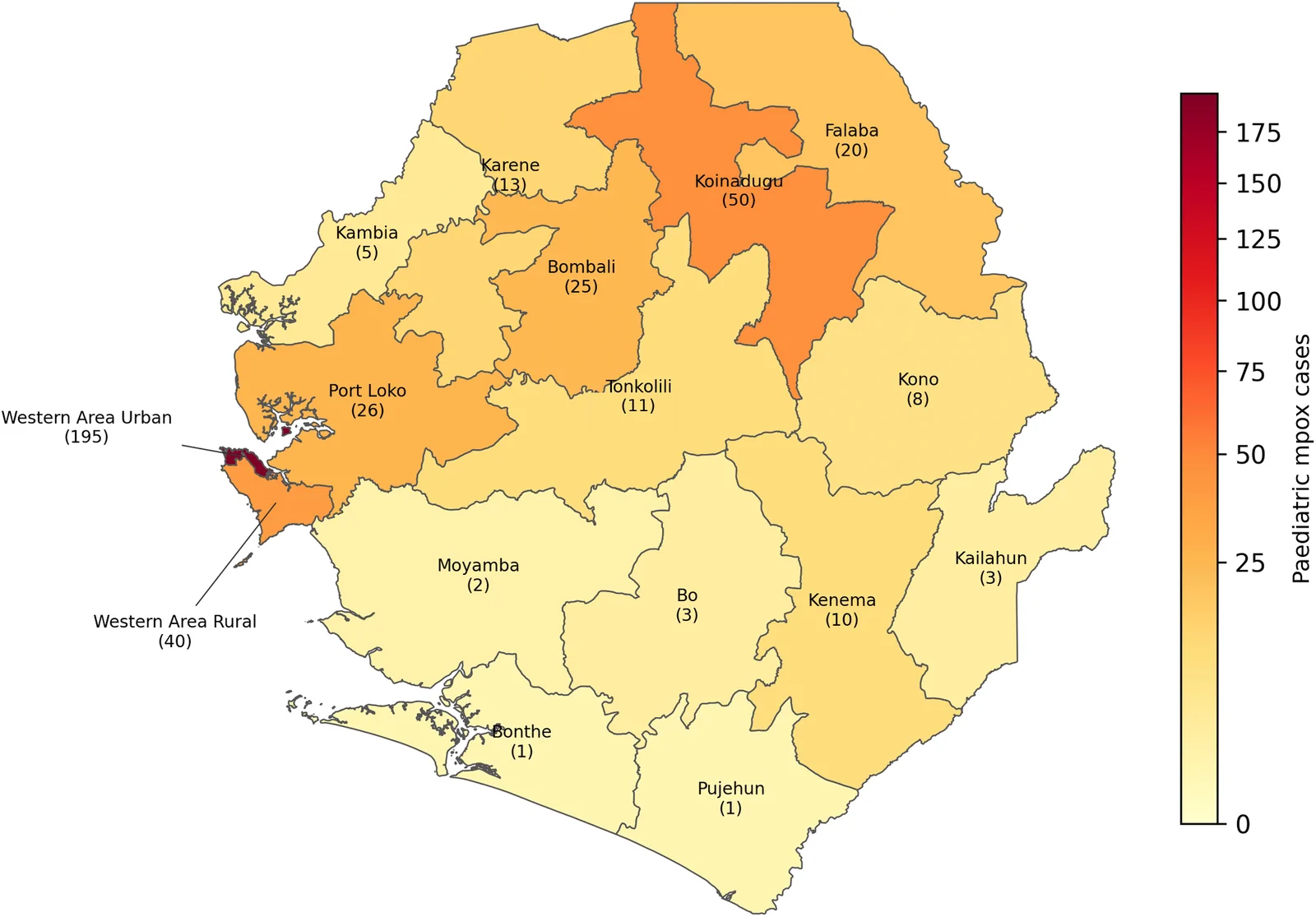
District distribution of paediatric mpox cases, Sierra Leone, 2024 to 2025. Labels and shading show notified case counts; the map does not depict incidence, risk, or transmission route. Administrative boundaries were obtained from the Humanitarian Data Exchange (https://data.humdata.org/dataset/cod-ab-sle; CC BY 3.0 IGO).

### Data completeness

Sex was recorded for all 415 children and district for 413 (99.5%). Care location was recorded for 285 (68.7%), final outcome for 261 (62.9%), and HIV status for 76 (18.3%). Table 4 shows corresponding completeness among cases aged 15 years or older.

**Table 4:** Completeness of selected surveillance fields in children and cases aged 15 years or older.

Field	Children younger than 15 years	Cases aged 15 years or older
Sex	415/415 (100.0)	5,051/5,051 (100.0)
Residential district	413/415 (99.5)	5,041/5,051 (99.8)
First facility-contact date	415/415 (100.0)	5,051/5,051 (100.0)
Care location	285/415 (68.7)	3,861/5,051 (76.4)
Final outcome (alive/dead)	261/415 (62.9)	3,610/5,051 (71.5)
HIV status	76/415 (18.3)	9,18/5,051 (18.2)

Note: Completeness is shown as n/N (%). HIV status refers to records with an available positive or negative value. MVA-BN vaccination completeness is reported separately for eligible children aged 12–14 years.

### Patient charts

Charts were retrieved for 162/216 children with recorded hospital care (75.0%), including all three who died; reasons for non-retrieval were not consistently documented. Admission indication was available in 119 charts: isolation or infection control alone in 48, clinical management plus isolation in 62, and clinical management alone in 9. Among all 162 charts, lesion burden was documented in 104, mucosal or ocular involvement in 27, hydration or nutritional status in 58, and treatment in 123. Median length of stay was 6 days (IQR 4–9) among 137 children with admission and discharge dates.

### Documented exposures

Outbreak-investigation forms were retrieved for 331/415 children (79.8%); 246 contained enough information to assign a primary exposure category. Household or caregiving contact was documented in 141/246 (57.3%), 48 (19.5%) had no single predominant exposure identified, 39 (15.9%) reported school, peer, or community contact, 12 (4.9%) reported animal, wildlife, or bushmeat exposure, and 6 (2.4%) reported health-care contact. Eight of the 12 animal-related records came from Koinadugu or Falaba. These categories describe reported exposures, not transmission routes.

### Genital and perianal lesions

After deduplicating the vaginal and perianal fields, 55/415 children had documented genital or perianal lesions. Charts or investigation forms were retrieved for 43/55. Of these 43 records, 26 (60.5%) documented a child-protection assessment or referral decision, 9 (20.9%) a referral to social-work or child-protection services, and 3 (7.0%) a forensic or medico-legal assessment. Records were retrieved for 18/22 girls aged 10–14 years with the ‘vaginal lesions’ field marked; 13/18 documented an assessment or referral decision and 6/18 a referral. The records did not establish sexual contact, sexual abuse, or another route of acquisition.

## Discussion

The shift from three sporadic paediatric cases of mpox-like illness reported from 2017 through 2024 to a rapid rise in notifications during 2025 shows that children formed a clear component of Sierra Leone’s clade IIb outbreak. Children younger than 5 years were the largest paediatric group. The paediatric proportion exceeded the 1.3% reported in the 2022 multicountry outbreak but remained below the 2024 estimate across African Union member states [5,10]. A Ugandan study also found the highest attack rate among children younger than 5 years [12]. Differences among settings likely reflect transmission context, population structure, care-seeking, testing, surveillance practice, and age definitions.

Temporal and geographic patterns identify priorities for field investigation, not routes of transmission. The paediatric curve rose during the national outbreak, but temporal concurrence does not identify who infected whom. Western Area Urban recorded the most cases, while Koinadugu and Falaba had high crude notified rates. These rates applied one national age proportion to every district; if northern populations were younger, their paediatric denominators were underestimated and rates overestimated. District-specific age denominators and testing data are needed before interpreting geographic differences as incidence or risk.

Household or caregiving contact was the most common documented exposure, but forms rarely captured linked-case identifiers, timing, contact testing, or competing exposures consistently. School, community, health-care, and animal-related contacts were also documented, including animal-related exposures in Koinadugu and Falaba. Prospective household and community contact studies and standardised animal-exposure questions are needed; these records do not establish a dominant transmission route.

Hospital care did not necessarily indicate severe disease because admission often served isolation and infection control. Fatal-case records described delayed presentation, poor feeding or dehydration, secondary bacterial infection, and malnutrition, but severity and outcome data were incomplete. National records should distinguish admission purpose and standardise documentation of severity, treatment, complications, and recovery.

Vaccination data were interpretable only among age-eligible cases. The campaign targeted selected groups aged 12 years or older, yet status was missing for half of eligible paediatric cases. The data could not distinguish eligibility, programme availability, and uptake or estimate coverage or effectiveness. Future records should capture vaccine product, dose, indication, eligibility, and timing. WHO’s 2024 position paper supports off-label MVA-BN vaccination of children during outbreaks when expected benefits exceed potential risks [18]. By September 2026, European authorisation for MVA-BN (Imvanex) included children aged 2 years and older, supported by paediatric immunogenicity data [23]. Given the 188 children younger than 5 years in this cohort, Sierra Leone’s future guidance should define eligibility for exposed younger children under applicable national authorisation, including off-label use where appropriate, with prospective safety and effectiveness monitoring [18,23]. HIV status was also sparse; complete, confidential documentation is needed when clinically indicated, particularly given the excess mortality among people living with HIV in the companion national analysis [24].

The DHIS2 ‘vaginal lesions’ field was marked more often among girls aged 10–14 years than younger girls but did not specify anatomy. Genital or perianal lesions do not identify the route of infection. Plausible explanations include disseminated disease, transfer from another body site, close caregiving contact, contaminated materials, sexual contact, sexual abuse, or recording error. The reviewed records could not distinguish among them. Clinicians should take an age-appropriate exposure history and seek child-protection or medico-legal assessment when the history or examination raises concern for abuse.

### Generalisability

The findings apply most directly to settings with passive, laboratory-supported surveillance during sustained clade IIb transmission. Paediatric case proportions, care patterns, and documented exposures may differ with population age structure, testing access, contact patterns, admission policy, and vaccination strategy.

### Strengths and limitations

The national cohort and supplementary chart and investigation-form review supported description by person, time, and place and added clinical and exposure context. Limitations arose from emergency surveillance. Passive case finding likely missed mild infections and children who did not seek care. An unmarked checkbox could indicate absence or non-entry. Record retrieval was incomplete, and exposure histories were not collected under a uniform analytic protocol. Outcomes, HIV status, severity, treatment, recovery, linked contacts, and referral decisions were incompletely documented. District rates relied on provisional total populations and a national age projection rather than district-specific paediatric denominators. These limitations preclude causal inference and restrict generalisability beyond settings with similar surveillance and care-seeking patterns.

## Conclusions

Children were not spared during Sierra Leone’s clade IIb outbreak. Mpox response plans should include children in surveillance, contact follow-up, clinical care, and vaccination assessment. National DHIS2 surveillance should include standardised fields for epidemiological links, clinical outcomes, MVA-BN eligibility and vaccination status, and documented exposure settings, supported by detailed outbreak-investigation forms. Prospective contact studies are needed to define paediatric transmission pathways and guide age-appropriate prevention.

## Supporting information

S1 Fig Cohort accounting for the national paediatric mpox surveillance analysis, Sierra Leone, 2024–2025. The figure begins with 5,468 cases meeting the national surveillance case definition, then shows the 5,466 cases with recorded age and the 415 paediatric cases by final classification.
(TIF)

S1 Data Aggregate values supporting the tables and figures. The workbook contains cohort accounting, the historical contextual count, table numerators and denominators, chart and outbreak-investigation summaries, weekly counts for Fig 1, and district counts and denominator calculations for Fig 2 and Table 3. Individual hospital-stay durations underlying the median and interquartile range are included in the restricted-access request. The workbook contains no individual-level patient records.
(XLSX)

S1 Checklist STROBE checklist for this retrospective national descriptive surveillance and outbreak-investigation study. (DOCX)
