# Supplementary material for: Paediatric mpox during Sierra Leone’s 2024 to 2025 clade IIb outbreak: A national surveillance and outbreak-investigation study of 415 children
Source: PLoS Negl Trop Dis. 2026 Oct 7;20(10):e0014785. doi: 10.1371/journal.pntd.0014785 (PMC13674859; doi:10.1371/journal.pntd.0014785)
Supplement: Supplementary file 1 [file pntd.0014785.s003.docx]

S1 Checklist

**Paediatric mpox during Sierra Leone's 2024 to 2025 clade IIb outbreak: a national surveillance and outbreak-investigation study of 415 children**

Manuscript ID: PNTD-D-26-00967R2 | Production revision, 2 October 2026 | Reference file: PNTD-D-26-00967R2_FTC.docx. Locations refer to section headings and tables in the final manuscript text; page numbers may change during production.

*Funding information is entered in the PLOS Financial Disclosure field, as required by the journal.*

| **Study design** | Retrospective national descriptive epidemiological study with supplementary review of available patient charts and outbreak-investigation forms |
| --- | --- |
| **National data source** | NPHA DHIS2 mpox surveillance database: 5,468 eligible records; 5,466 with age; 415 children younger than 15 years |
| **Supplementary records** | 162/216 hospital-care charts retrieved, including all three deaths; 331/415 outbreak-investigation forms retrieved |
| **Reporting framework** | STROBE for observational studies |

| **Item** | **Topic** | **STROBE recommendation** | **Location in final manuscript** | **Evidence in the manuscript** |
| --- | --- | --- | --- | --- |
| 1 | Title and abstract | State the study design in the title or abstract and provide a balanced summary. | Title page; Abstract | The title page identifies the national surveillance and outbreak-investigation study and lists the authors and affiliations, as required by PLOS. The structured abstract names the retrospective descriptive design and reports the principal person, time, place, clinical, exposure, and vaccination findings. |
| 2 | Background and rationale | Explain the scientific background and rationale. | Introduction | The Introduction contrasts earlier African paediatric burden with the adult-dominated 2022 outbreak, summarises contemporary African evidence, reports three sporadic paediatric cases of mpox-like illness in Sierra Leone from 2017 through 2024, and explains why the 2025 outbreak provides an informative national dataset. |
| 3 | Objectives | State specific objectives and prespecified hypotheses when applicable. | Introduction, final paragraph; Methods, Statistical analysis | The objective is to describe paediatric mpox by person, time, and place and to summarise clinical features, care, outcomes, vaccination records, documented exposures, and data completeness. The Methods state that the prespecified objective was descriptive and that no aetiological hypothesis was specified. |
| 4 | Study design | Present key elements of the study design early. | Methods, Study design, setting, and cohort | After the Ethics Statement, the Methods describe a retrospective national descriptive study using the national DHIS2 database, historical NPHA surveillance summaries for temporal context, and supplementary chart and outbreak-investigation-form review. |
| 5 | Setting | Describe setting, locations, and relevant dates. | Methods, Study design, setting, and cohort | The manuscript identifies Sierra Leone, the NPHA national surveillance system, the 2017–2024 historical context, the 2024–2025 outbreak, the January and December 2025 emergency milestones, and the 1 July 2026 database freeze. |
| 6 | Participants | Describe eligibility criteria, sources, and methods of participant selection. | Methods, Study design, setting, and cohort; Variables and supplementary record review; Results, Patient charts; Documented exposures; S1 Fig | All records with final national classification as laboratory-confirmed, probable, or clinician-diagnosed were eligible. Two records lacked age. The paper defines paediatric age groups and states the chart and form populations sought and the numbers retrieved. |
| 7 | Variables | Define outcomes, exposures, predictors, confounders, and effect modifiers. | Methods, Variables and supplementary record review; Exposure and vaccination definitions; Statistical analysis | Person, time, and place variables are defined. The Methods specify the mortality denominator, checkbox interpretation, chart variables, exposure categories, child-protection variables, MVA-BN eligibility, and district-rate denominators and the source, licence, and software used for the district map. |
| 8 | Data sources and measurement | For each variable, give data sources and assessment methods. | Methods, Study design, setting, and cohort; Variables and supplementary record review; Statistical analysis | The Methods distinguish historical NPHA summaries, DHIS2, patient charts, and outbreak-investigation forms and state how dates, case classification, symptoms, lesion fields, clinical variables, exposure categories, vaccination, and district denominators were measured. |
| 9 | Bias | Describe efforts to address potential sources of bias. | Methods, Variables and supplementary record review; Statistical analysis; Table 2 note; Discussion, Strengths and limitations | The paper codes unmarked checkboxes as not documented, limits supplementary analyses to retrieved records, uses field-specific denominators, labels district rates as notified burden, protects fatal-case privacy, and discusses passive surveillance, missing fields, record retrieval, and age-denominator bias. |
| 10 | Study size | Explain how the study size was determined. | Methods, Study design, setting, and cohort; Results, Person; S1 Fig | The study includes the full frozen national cohort meeting the case definition. No sample-size calculation was used. S1 Fig reports the complete cohort flow. |
| 11 | Quantitative variables | Explain how quantitative variables were handled and any groupings. | Methods, Study design, setting, and cohort; Variables and supplementary record review; Statistical analysis | Age was grouped as 0–4, 5–9, and 10–14 years. Time was epidemiological week. Continuous values are summarised with medians and IQRs; district rates use stated denominator assumptions, and the district map source and software are reported. |
| 12 | Statistical methods | Describe statistical methods, subgroup analyses, missing data, and sensitivity analyses. | Methods, Statistical analysis; Results, Data completeness; Table 4 | The analysis reports counts, field-specific percentages, medians with IQRs, an exact binomial confidence interval for mortality, the epidemic curve, and crude district notification rates. Missingness is displayed in Table 4. No inferential child-versus-older-age testing is used. |
| 13 | Participants | Report numbers at each stage of the study. | Results, Person; Patient charts; Documented exposures; Genital and perianal lesions; S1 Fig | The paper reports 5,468 eligible records, 5,466 with age, 415 children, 162/216 retrieved charts, 331/415 retrieved forms, 246 classifiable forms, and record retrieval for the genital and perianal lesion review. |
| 14 | Descriptive data | Give participant characteristics, exposures, and missing data. | Results, Person; Place; Data completeness; Patient charts; Documented exposures; Tables 1, 3 and 4 | Table 1 gives age-specific demographic and clinical characteristics. Table 3 gives district counts and rates. Table 4 gives field completeness. Supplementary Results report chart and exposure data. |
| 15 | Outcome data | Report outcome event numbers or summary measures. | Results, Person; Table 2 | The paper reports three deaths among 261 children with recorded outcomes, the exact confidence interval, onset-to-care intervals, and privacy-protected clinical summaries of the three deaths. |
| 16 | Main results | Give unadjusted estimates and precision; report adjusted estimates where applicable. | Results, Person; Time; Place; Patient charts; Tables 1 to 4 | The study reports descriptive counts and percentages with field-specific denominators, medians and IQRs, an exact mortality confidence interval, historical and outbreak timing, weekly counts, and crude district rates. Adjusted estimates were not applicable to the descriptive objective. |
| 17 | Other analyses | Report subgroup and other analyses. | Results, Person; Time; Place; Documented exposures; Genital and perianal lesions | The manuscript reports MVA-BN status in the age-eligible subgroup, the historical temporal context and weekly epidemic curve, district notified rates, documented exposure categories, and age-specific genital-lesion fields and child-protection documentation. |
| 18 | Key results | Summarise key results with reference to objectives. | Discussion, opening paragraphs | The Discussion interprets the transition from sporadic reports to the 2025 rise, paediatric involvement, the person-time-place pattern, documented exposures, hospital care, vaccination, HIV missingness, and genital and perianal lesion documentation in relation to the objective. |
| 19 | Limitations | Discuss limitations, direction of bias, and imprecision. | Discussion, Strengths and limitations; Place interpretation | The limitations address passive ascertainment, checkbox ambiguity, incomplete retrieval, non-standardised exposure histories, missing clinical and outcome fields, and overestimation of district rates where child populations exceed the national projection. |
| 20 | Interpretation | Give a cautious interpretation considering objectives, limitations, multiplicity, and other evidence. | Discussion; Conclusions | The Discussion and Conclusion distinguish documented exposures from transmission routes, hospital care from severity, vaccination documentation from coverage, and notified rates from incidence. The vaccination paragraph addresses WHO outbreak guidance, current European authorization for MVA-BN, and national eligibility guidance for exposed younger children with prospective safety and effectiveness monitoring. |
| 21 | Generalisability | Discuss the generalisability of the results. | Discussion, Generalisability | A dedicated Generalisability subsection identifies the surveillance settings to which the findings apply most directly and the factors that may change paediatric case proportions, care patterns, and documented exposures. |
| 22 | Funding | State the source of funding and the role of funders. | PLOS Financial Disclosure field | Submission-form statement: “The authors received no specific funding for this work.” PLOS instructs authors to enter funding information in the Financial Disclosure field rather than in the manuscript file. |
