# Supplementary material for: Paediatric mpox during Sierra Leone’s 2024 to 2025 clade IIb outbreak: A national surveillance and outbreak-investigation study of 415 children
Source: PLoS Negl Trop Dis. 2026 Oct 7;20(10):e0014785. doi: 10.1371/journal.pntd.0014785 (PMC13674859; doi:10.1371/journal.pntd.0014785)
Supplement: Supplementary file 2 [file pntd.0014785.s004.docx]

**Paediatric mpox during Sierra Leone’s 2024-2025 clade IIb outbreak: a national surveillance study of 276 children.**

In this study the authors describe mpox in children affected during the Clade IIb outbreak in Sierra Leone, probably the largest cohort of children described in west Africa to date. This topic is highly relevant for the global health community as several important epidemiological questions remain unanswered, particularly regarding paediatric mpox. Although the study is based on retrospectively analysed surveillance data with its inherent limitations, it provides valuable and timely information for the field. As the authors already described the limitations of the study, I only have minor comments.

1. I think the title could be clearer by describing the nature of the study. For example, adding “retrospective analysis” of national surveillance data to the current title.
2. In the abstract, the end of the background section could be followed by the study objective of this analysis to guide the reader through a smooth transition into the methodology and principal findings section.
3. “eligible” as used in the manuscript already features in the abstract. It would therefore be useful to already provide a brief definition at this level, for the current abstract to be a stand-alone summary.
4. In line 27 of the abstract, a statement appears to be strangely written. *“Falaba contributed 6.9% but 0.7%”* as a full sentence doesn’t seem to be OK. I understand that this is in line with the information from the preceding sentence, yet I would propose to rewrite it accordingly
5. Following comment 4 above, may I also ask that the authors consistently indicate numerators and denominators for all percentages indicated? This should be done consistently across the entire manuscript?
6. Line 44-45, summary, the authors wrote *“A practical response should start with household mapping. When an adult case is found, children in the same household should be identified, monitored, protected from lesion contact and considered for post-exposure vaccination where vaccine is available.”* Although such a strategy could support outbreak response, it is unclear how the analysis presented in this study supports this conclusion. In my opinion, the authors should focus on describing from the data available. The message could be a bit softened and suggestive rather than affirmative!
7. Line 44–45 (Summary): The recommendation that outbreak response should begin with household mapping and that children in affected households should be identified, monitored, protected from lesion contact, and considered for post-exposure vaccination appears stronger than what is directly supported by the analyses presented in this manuscript. While these measures may be reasonable public health interventions, the study is primarily descriptive and does not appear to evaluate household transmission dynamics, contact tracing strategies, or intervention effectiveness. I would therefore suggest softening this statement and framing it as a potential implication of the findings rather than a definitive recommendation. The conclusions should remain closely aligned with the evidence generated by the study.
8. The comment above also applies to the main manuscript text area (line 231-239). I would therefore also suggest softening these statements and stick to the evidence provided by the authors.
9. Line 53–54: The statement that historical data from clade I settings “cannot define paediatric risk during clade IIb epidemics” appears overly categorical. Consider softening this statement and clarifying which aspect of pediatric risk (e.g., infection risk, disease severity, or outcomes) may not be directly generalizable to clade IIb epidemics.
10. Line 60: Children may face exposure through household care, shared bedding, bathing...? Do the authors mean the sharing of items such as towels? Bathing itself sounds very specific.
11. Line 69-70: The statement that the study "identified lessons for household contact management, paediatric vaccination policy and rural preparedness" appears stronger than what is directly supported by the analyses presented. The study provides valuable descriptive epidemiological data on pediatric mpox cases; however, it does not appear to formally evaluate household contact management strategies, vaccination policies, or preparedness interventions. Consider reframing these points as potential public health implications or areas for future investigation rather than as lessons identified by the study itself.
12. Line 81: (Case definitions and analytic cohort) I would suggest that the authors first state the national/general case definition(s) used during the outbreak before stating the case definitions used for the analytical cohort of this study. That way, it’s easier to immediately see the discrepancy between the denominators even before this is explained in the following lines 86-90
13. Line 83: The phrase *"Confirmed cases recorded polymerase chain reaction evidence of mpox infection"* is somewhat unclear and does not provide a formal case definition. I suggest clearly stating the surveillance case definitions used in the study. For example: *"A suspected case was defined as..." and "A confirmed case was defined as a suspected case with laboratory confirmation of mpox virus infection by PCR."* This would make the methods easier to follow and ensure consistency with standard outbreak reporting practices.
14. Results section, as I already mentioned above but I think it may be worth mentioning again here. I would suggest that numerators and denominators accompany percentages even in text. So far, the authors have tried to do so in tables, yet somewhat inconsistently.
15. For the broad scientific audience, I would suggest defining what criteria was used to decide on a child receiving either home isolation or hospital care somewhere in the methods section. Could be a short paragraph for example on public health response strategy?
16. Majority of the children received hospital care. Was this decided based on severity? In line with my comment above, kindly mention criteria used.
17. Table 1, I am not sure if it is relevant to state the median age for each age category. I would suggest keeping only the overall median.
18. Table 1, the denominators dont completely add up. n=131 in the 0-4 year age category. Yet deaths 2/130? Was there missing data? Also, for type of care denominator is 124. I would suggest adding missing/unreported information for each of the variables. Again, I would like to reiterate my comment about including all numerators and denominators across the entirety of the manuscript
19. Was the outcome data available for everyone whether they were under home isolation or hospital care?
20. Line 147: *“It does not prove household transmission.”* Shouldn’t this statement be reserved for the discussion?
21. Line 169 I would suggest to add “*rural*” in brackets after Koainadugu, Falaba, and Karene? Also, in the table? Easier for the readers to conpare wth the Western Area urban.
22. Where mentioned in the study, was vaccination always post-exposure? It would be good to clarify that children were not vaccinated as part of a vaccination campaign but rather post-exposure.
23. Line 200-203: The interpretation of age distribution appears to be too inferential. While the higher burden among children aged 0–4 years may be consistent with increased household exposure, the manuscript does not present data on specific exposure routes or caregiving activities. The proposed mechanisms (e.g., bathing, feeding, sleeping) are not supported by the analyses presented so I would suggest being toned down or clearly presented as hypotheses rather than inferred pathways. If the authors would like to keep the idea, I would suggest rephrasing such as *“The higher incidence among children aged 0–4 years is consistent with increased exposure within household settings, where close and prolonged caregiver contact is common. However, specific transmission pathways could not be assessed in this study.”*
24. Lines 231-239: from the statement *“Pediatric mpox guidance should be ready before the next outbreak... ”* and everything else that follows in this paragraph shifts the tone of the manuscript from scientific descriptive to a time-bound and imperative, assertive message sort of implying a system-level readiness assessment was conducted by the study. While these are important and reasonable considerations, they are not supported by the manuscript. I would therefore suggest distinguishing more clearly between observed findings and broader implications, and reframing these statements as hypotheses or areas for future operational and clinical guidance development.
