# Supplementary material for: Paediatric mpox during Sierra Leone’s 2024 to 2025 clade IIb outbreak: A national surveillance and outbreak-investigation study of 415 children
Source: PLoS Negl Trop Dis. 2026 Oct 7;20(10):e0014785. doi: 10.1371/journal.pntd.0014785 (PMC13674859; doi:10.1371/journal.pntd.0014785)
Supplement: Supplementary file 3 [file pntd.0014785.s006.docx]

Response to Editors and Reviewer

Manuscript ID: PNTD-D-26-00967R1

**Revised title: Paediatric mpox during Sierra Leone's 2024 to 2025 clade IIb outbreak: a national surveillance and outbreak-investigation study of 415 children**

Date: 16 July 2026

Dear Dr Simons, Dr Holbrook, Dr Kamhawi, Dr Brindley, and Reviewers,

Thank you for the detailed review. We rebuilt the analysis around the complete national surveillance cohort and revised the manuscript as a self-contained retrospective descriptive epidemiological study. The paper now follows a person, time, and place structure, includes a weekly epidemic curve, and uses patient charts and outbreak-investigation forms only for the clinical and exposure information available in those records. It also distinguishes three sporadic paediatric cases of mpox-like illness reported from 2017 through 2024 from the rapid rise in 2025. We removed unsupported causal claims, nonessential hypothesis tests, the compositional district ratio, and repeated statements across sections.

Section, table, and figure references below refer to the final clean manuscript in this package.

# Principal revisions

- Rebuilt the cohort from the national case-classification field: 5,468 eligible records, 5,466 with age, and 415 children younger than 15 years. S1 Fig documents every stage.
- Organised the study and Results by person, time, and place, added a weekly paediatric epidemic curve as Fig 1, and clarified the transition from three sporadic paediatric mpox-like illnesses reported during 2017 through 2024 to the rapid 2025 rise.
- Removed the child-versus-older-age hypothesis-test table, p values, risk ratios, the Wilcoxon comparison, and the case-composition ratio. The analysis is descriptive.
- Reported final case classification by paediatric age group and placed the 411/415 paediatric laboratory-confirmation count in the context of the 5,445/5,468 national count.
- Reported field completeness explicitly and used field-specific denominators throughout.
- Condensed the Methods into five focused subsections while retaining the chart and outbreak-investigation details needed to reproduce the admission, exposure, and fatal-case analyses. We also clarified the prespecified descriptive objective, stated that no aetiological hypothesis was specified, and identified the supplementary chart and investigation-form analyses as exploratory.
- Restricted MVA-BN analysis to children aged 12 to 14 years and interpreted the records as documentation among age-eligible cases, not coverage, access, or effectiveness.
- Reported genital and perianal lesion fields and child-protection documentation directly, without inferring sexual contact or abuse from lesion location.
- Updated the ethics and consent statement, aggregate S2 Data workbook, Data Availability statement, figures, STROBE checklist, and submission-form text.

# Responses to the Additional Editor Comments

## 1. Data integrity and denominator reconciliation

**Comment:** *Reconcile the public 5,442 confirmed-case count with the analytic denominator, explain the earlier loss of records, provide a cohort flow diagram, and account for varying denominators.*

**Response:** The earlier 4,140 denominator represented an incomplete analytic extraction and is no longer used. We rebuilt the cohort directly from the populated national DHIS2 case-classification field. The frozen 1 July 2026 extract contained 5,445 records with final laboratory-confirmed classification, 20 probable records, and 3 clinician-diagnosed records, for 5,468 eligible cases. The Ministry of Health outbreak-end report and the analytic extract were different data snapshots; the extract contained three more records with final laboratory-confirmed classification. We do not assign an undocumented reason to that difference. No eligible record was excluded from the revised cohort. Two records lacked age, leaving 5,466 for age-specific analyses and 415 children. S1 Fig shows the flow. Tables use field-specific denominators, Table 4 reports completeness, and S2 Data supplies the supporting numerators, denominators, formulas, weekly counts, district counts, and automated checks.

Location: Methods, Study design, setting, and cohort; Results, Person and Data completeness; Table 4; S1 Fig; S2 Data.

## 2. Spatial interpretation and hospital care

**Comment:** *Avoid interpreting a compositional district metric as incidence or risk, and distinguish hospital isolation from clinical severity.*

**Response:** We removed the paediatric case-composition ratio and its inferential interpretation. The Place section reports district counts, proportions among records with known district, and crude notified rates calculated from provisional district populations. We corrected the national projected proportion younger than 15 years to 40.1% and applied it uniformly because district-specific age structures were unavailable. The Methods, table note, figure caption, Discussion, and limitations state that the rates describe notified burden rather than incidence or risk and may be overestimated where the true child population exceeds 40.1%. Patient-chart review separates isolation or infection-control care from clinical management, and the limitations state that passive surveillance probably missed mild cases and children who did not reach care.

Location: Methods, Statistical analysis; Results, Place and Patient charts; Discussion, geographic and hospital-care paragraphs; Strengths and limitations; Fig 2; Table 3.

## 3. Diagnostic robustness and HIV

**Comment:** *Report confirmation by paediatric age group and address HIV status as a clinical confounder and source of missingness.*

**Response:** Table 1 reports laboratory-confirmed classification for each paediatric age group: 187/188 children aged 0–4 years, 120/121 aged 5–9 years, and 104/106 aged 10–14 years. Its note reports one, one, and two probable cases across these groups; no paediatric case was clinician-diagnosed. The Results place the 411/415 paediatric count alongside the national count of 5,445/5,468 and do not present laboratory confirmation as a distinctive paediatric finding. HIV status is reported with its denominator, Table 4 shows completeness in children and cases aged 15 years or older, and the Discussion treats sparse HIV data as a limitation.

Location: Methods, Study design, setting, and cohort and Variables and supplementary record review; Results, Person and Data completeness; Tables 1 and 4; Discussion, vaccination and HIV paragraphs.

## 4. Transmission, vaccination, genital lesions, and descriptive framing

**Comment:** *Keep transmission conclusions within the limits of passive surveillance, account for MVA-BN eligibility, address genital lesions and child-protection information, and retain a descriptive epidemiological framing.*

**Response:** The objective now states that the study describes paediatric mpox by person, time, and place. Exposure categories describe documented histories in retrievable forms and do not assign route of infection. Household or caregiving, school or community, health-care, and animal-related contacts remain hypotheses for prospective study. The vaccination analysis is restricted to children aged 12–14 years, the age-eligible paediatric band in this cohort. For genital and perianal lesions, the Methods state exactly what was abstracted: assessment of possible sexual contact or abuse and any social-work, child-protection, forensic, or medico-legal review. The Results report the available documentation. The Discussion states sexual and nonsexual explanations directly and explains that lesion location does not identify acquisition route. The Abstract conclusion and main Conclusion state implications rather than repeat results.

Location: Abstract; Methods, Variables and supplementary record review, Exposure and vaccination definitions, and Statistical analysis; Results, Person, Documented exposures, and Genital and perianal lesions; Discussion; Conclusions.

# Responses to Reviewer 2 Major Comments

## Major Comment 1. Geographic analysis

**Comment:** *The district ratio is compositional and cannot establish increased paediatric risk or preferential household transmission.*

**Response:** We agree and removed the ratio. Residence results use 413 paediatric and 5,041 older-age records with known district. Table 3 reports case counts and crude notified rates derived from provisional population denominators. The text labels the estimates as notified burden and describes the direction of bias introduced by a uniform national age proportion. Geographic findings now identify districts for field investigation; they do not estimate incidence, risk, or transmission route.

Location: Methods, Statistical analysis; Results, Place; Discussion, geographic paragraph; Fig 2; Table 3.

## Major Comment 2. MVA-BN eligibility and supply context

**Comment:** *Name the vaccine, account for age eligibility and constrained supply, avoid pooled access claims, and address possible miscoding.*

**Response:** The Methods name MVA-BN, describe Sierra Leone's targeted single-dose campaign for selected groups aged 12 years or older, and restrict the paediatric analysis to children aged 12–14 years. Entries in younger children were excluded because the sources could not distinguish routine childhood immunisation, exceptional MVA-BN use, or miscoding. The Results report 27/54 eligible cases with a recorded status and six vaccinated. The manuscript does not interpret these data as coverage, access, or effectiveness and identifies the missing product, dose, indication, eligibility, and timing fields.

Location: Methods, Exposure and vaccination definitions; Results, Person; Discussion, vaccination paragraph.

## Major Comment 3. Genital and perianal lesions and child protection

**Comment:** *Present the age pattern, clarify exposure histories, and report child-protection, social-work, forensic, or medico-legal assessment.*

**Response:** Table 1 reports the DHIS2 “vaginal-lesion” field separately for girls aged 0–4, 5–9, and 10–14 years. The Methods state that the field did not distinguish external vulval, perineal, or intravaginal location and describe the direct abstraction of possible sexual contact or abuse assessments and referrals. After deduplication of vaginal and perianal fields, 55 children had genital or perianal lesion documentation; records were retrieved for 43, of which 26 documented a child-protection assessment or referral decision, 9 documented social-work or child-protection referral, and 3 documented forensic or medico-legal assessment. The paper does not infer sexual exposure or abuse from lesion location.

Location: Methods, Variables and supplementary record review; Results, Person and Genital and perianal lesions; Discussion, genital and perianal lesion paragraph.

## Major Comment 4. Clinical description and targeted chart abstraction

**Comment:** *Clarify the clinical fields available in DHIS2 and add chart abstraction for hospitalised or fatal paediatric cases where feasible.*

**Response:** The Methods list the DHIS2 variables and the national clinical fields that were missing or incomplete. We sought charts for every child with recorded hospital care and all three deaths. We retrieved 162/216 hospital-care charts, including all three deaths. Chart abstraction covered admission purpose, lesion burden and distribution, mucosal or ocular involvement, hydration and nutrition, comorbidities, treatment, dates, complications, outcomes, and death circumstances. The Results report admission context, completion of key clinical fields, and length of stay.

Location: Methods, Variables and supplementary record review; Results, Patient charts; Table 2.

## Major Comment 5. Clinical context for the three deaths

**Comment:** *Provide concise clinical descriptions of each fatal case and keep the interpretation cautious.*

**Response:** Table 2 reports grouped age and sex, onset-to-care interval, admission context, clinical findings, HIV, nutrition and comorbidity documentation, and treatment or complications. The table note identifies the source records and explains their limits. Exact dates, exact ages, and district details were omitted to reduce re-identification risk. The text reports three deaths among 261 children with recorded outcomes and an exact 95% confidence interval. It does not infer mortality predictors or mortality among children with missing outcomes.

Location: Results, Person; Table 2.

## Major Comment 6. Laboratory confirmation by age group

**Comment:** *Report confirmation categories within each paediatric age group and address possible childhood-rash misclassification.*

**Response:** Table 1 reports the age-specific laboratory-confirmed counts. Its note gives the probable counts by age group: one among children aged 0–4 years, one among those aged 5–9 years, and two among those aged 10–14 years. No paediatric case was clinician-diagnosed. This addresses the concern about reliance on clinical diagnosis in young children. The paper also places the paediatric count beside the full national cohort and does not portray laboratory confirmation as a surprising paediatric feature.

Location: Methods, Study design, setting, and cohort; Results, Person; Table 1.

# Responses to Reviewer 2 Minor Comments

## Hospital care and isolation policy

**Response:** The Results separate isolation or infection-control care, clinical management plus isolation, and clinical management alone. The Discussion states that hospital care cannot serve as a severity endpoint.

Location: Results, Patient charts; Discussion, hospital-care paragraph.

## Comparison with the 1.3% multicountry paediatric proportion

**Response:** The Introduction and Discussion now attribute between-setting differences to transmission context, population structure, surveillance, testing, care-seeking, and age definitions, rather than to age cut-offs alone.

Location: Introduction; Discussion, opening paragraph.

## Effect estimates, p values, and multiplicity

**Response:** We removed the exploratory child-versus-older-age inferential table, p values, risk ratios, and multiple-comparison language. The analysis now reports descriptive counts, percentages, medians, and one exact confidence interval around the mortality proportion.

Location: Methods, Statistical analysis; Results throughout.

## Data completeness by age group and potential bias

**Response:** Table 4 reports completeness separately for children and cases aged 15 years or older for sex, district, facility date, care location, outcome, and HIV status. The limitations explain how incomplete fields and record retrieval may bias interpretation.

Location: Results, Data completeness; Table 4; Strengths and limitations.

## Onset-to-care comparison

**Response:** We removed the child-versus-older-age Wilcoxon comparison from the Methods and Results because it did not add value to the descriptive study. Onset-to-care intervals remain only in the privacy-protected descriptions of the three deaths.

Location: Results, Person; Table 2.

## Figure 2 interpretation

Response: The Fig 2 title and legend state that the map displays notified counts and does not show incidence, risk, or transmission route. The Methods and legend now identify the Government of Sierra Leone administrative boundary shapefiles distributed through the Humanitarian Data Exchange under the Creative Commons Attribution 3.0 IGO licence, and the Methods state that the map was created in R version 4.6.1.

Location: Results, Place; Fig 2.

## Author Summary and Conclusions

**Response:** The Author Summary distinguishes documented exposure from transmission route. The Abstract conclusion and main Conclusion state operational and research implications without repeating numerical results or presenting household transmission as proven.

Location: Abstract; Author summary; Conclusions.

# Journal and technical requirements

- The package includes a clean manuscript, a highlighted revised manuscript, this response letter, and a cover letter. The manuscript retains author names and affiliations on the title page because PLOS Neglected Tropical Diseases uses single-anonymized peer review; patient records remain de-identified.
- Fig1.tif and Fig2.tif are separate RGB TIFF files at 300 dpi with LZW compression and journal-compliant dimensions. The manuscript identifies the source and licence for the administrative boundary shapefiles used in Fig 2. S1_Fig.tif is supplied as Supporting Information.
- S2_Data.xlsx contains aggregate historical context, cohort, table, chart-review, outbreak-investigation, epidemic-curve, district, and denominator values. It contains no individual-level or synthetic patient records, and every automated consistency check returns PASS.
- The Data Availability statement identifies the Directorate of Policy, Planning and Research, Research Ethics Focal Person, as the non-author institutional access route and explains why individual-level surveillance, chart, and investigation records remain restricted.
- The Sierra Leone Ethics and Scientific Review Committee approved the secondary analysis under SLESRC-2025-049 and confirmed that individual informed consent was not required.
- The financial disclosure, competing-interests statement, and Data Availability text are reproduced in the internal submission checklist for accurate entry in Editorial Manager.
- The completed STROBE checklist gives final page references and supporting evidence for all 22 items.

We thank the editors and reviewer for comments that improved the epidemiological precision, clinical detail, privacy protections, and public-health value of the manuscript.

On behalf of all authors,

Eric Nzirakaindi Ikoona
National Public Health Agency, Freetown, Sierra Leone
Corresponding author
