# Supplementary material for: Paediatric mpox during Sierra Leone’s 2024 to 2025 clade IIb outbreak: A national surveillance and outbreak-investigation study of 415 children
Source: PLoS Negl Trop Dis. 2026 Oct 7;20(10):e0014785. doi: 10.1371/journal.pntd.0014785 (PMC13674859; doi:10.1371/journal.pntd.0014785)
Supplement: Supplementary file 4 [file pntd.0014785.s007.docx]

Response to the editor and reviewer

Manuscript PNTD-D-26-00967R2 | Second revision | September 10, 2026

Paediatric mpox during Sierra Leone's 2024 to 2025 clade IIb outbreak: a national surveillance and outbreak-investigation study of 415 children

Dear Dr Simons and Reviewer 2,

Thank you for the September 9, 2026 decision. We added three sentences on vaccination in younger children and updated the supporting reference. The response below compares district notification patterns and addresses the diagnostic-classification question. The tracked manuscript shows changes from the 415-child manuscript reviewed in this round. We also clarified the scope of restricted data access and aligned the institutional contact details across the submission files.

# Major comment on vaccination in younger children

**Comment: Add 2–3 sentences addressing off-label or expanded-eligibility MVA-BN vaccination in younger children during outbreaks, grounded in the WHO 2024 position paper.**

Response: The vaccination paragraph in the Discussion now includes:

WHO's 2024 position paper supports off-label MVA-BN vaccination of children during outbreaks when expected benefits exceed potential risks [18]. By September 2026, European authorization for MVA-BN (Imvanex) included children aged 2 years and older, supported by pediatric immunogenicity data [23]. Given the 188 children younger than 5 years in this cohort, Sierra Leone's future guidance should define eligibility for exposed younger children under applicable national authorization, including off-label use where appropriate, with prospective safety and effectiveness monitoring [18,23].

The WHO recommendation supports consideration of vaccination during outbreaks. The EMA update supplies the current European age indication and pediatric immunogenicity evidence. European authorization does not establish Sierra Leone campaign eligibility. The analysis therefore retains the historical campaign threshold of 12 years and does not estimate vaccine coverage or effectiveness.

Location: Discussion, vaccination paragraph; references 18 and 23. The companion national HIV-mpox reference is renumbered from 23 to 24.

# Minor comment on consistency between cohort versions

**Comment: Confirm whether district notification patterns and age-specific laboratory-confirmation proportions remain consistent after the cohort changed from 276 to 415 children.**

Response on geography: Western Area Urban recorded the largest paediatric count in both cohorts. Koinadugu and Falaba retained high crude notified rates. The direction of these findings persists, but the magnitude and rate ranking changed.

**Table A. District counts and crude paediatric notification rates in both cohort versions.**

| **District** | **Earlier cohort n (% of 276)** | **Earlier rate** | **Revised cohort n (% of 413)** | **Revised rate** |
| --- | --- | --- | --- | --- |
| Western Area Urban | 96 (34.8) | 39.5 | 195 (47.2) | 80.2 |
| Koinadugu | 45 (16.3) | 54.4 | 50 (12.1) | 60.5 |
| Falaba | 19 (6.9) | 28.5 | 20 (4.8) | 30.0 |
| Karene | 12 (4.3) | 10.3 | 13 (3.1) | 11.2 |
| Bombali | 24 (8.7) | 15.5 | 25 (6.1) | 16.1 |
| Port Loko | 23 (8.3) | 10.9 | 26 (6.3) | 12.3 |
| Western Area Rural | 20 (7.2) | 7.5 | 40 (9.7) | 15.1 |
| Other nine districts | 37 (13.4) | Not calculated | 44 (10.7) | Not calculated |

Rates are per 100,000 children. Both sets use the current Table 3 denominators: provisional 2021 district populations multiplied by 0.401. Earlier rates were calculated for this response from the district counts in the original 276-child manuscript; they were not reported in that manuscript. Revised percentages use 413 children with recorded district; two lack district. S2 Data supplies the revised counts and population calculations.

Western Area Urban rose from 39.5 to 80.2 per 100,000 and moved from second to first in the rate ranking, ahead of Koinadugu. Western Area Rural also approximately doubled, from 7.5 to 15.1. Koinadugu changed from 54.4 to 60.5 and Falaba from 28.5 to 30.0. The combined paediatric share from Koinadugu, Falaba, and Karene fell from 76/276 (27.5%) to 83/413 (20.1%). These comparisons describe changes in aggregate counts; they do not establish which individual records entered, left, or changed classification between extracts.

The manuscript retains its interpretation: these patterns identify priorities for field investigation. Passive ascertainment and the use of a national age proportion for every district prevent interpreting these notified rates as incidence or risk.

Location: Results, Place; Discussion, geographic paragraph; Table 3; Fig 2.

Response on diagnostic classification: In the revised cohort, laboratory confirmation is recorded for 187/188 children aged 0 to 4 years (99.5%), 120/121 aged 5 to 9 years (99.2%), and 104/106 aged 10 to 14 years (98.1%). The corresponding probable counts are one, one, and two. No child is classified as clinician-diagnosed. Table 1 therefore provides no indication of greater reliance on clinical-only diagnosis in the youngest age group within the revised cohort.

The original 276-child manuscript did not report laboratory-confirmation counts by age group. A numerical comparison with the original cohort cannot be verified from that manuscript. We therefore do not claim unchanged age-specific proportions between versions.

Location: Results, Person; Table 1 and its note; Methods, Study design, setting, and cohort.

# Journal requirement on data availability

We request editorial approval for restricted access to the individual-level surveillance records, patient charts, and outbreak-investigation forms. The separate request explains the confidentiality and child-protection restrictions and identifies a non-author institutional access route through the National Public Health Agency. The request explicitly includes the individual hospital-stay durations underlying the reported median and interquartile range. S2 Data supplies aggregate supporting values and directs requests for the underlying durations to the same institutional route. The data availability statement and institutional telephone number are consistent across the revised files. The reported results remain unchanged.

Thank you for considering the revision.

Eric Nzirakaindi Ikoona
Corresponding author
On behalf of the authors
