# Supplementary material for: Paediatric mpox during Sierra Leone’s 2024 to 2025 clade IIb outbreak: A national surveillance and outbreak-investigation study of 415 children
Source: PLoS Negl Trop Dis. 2026 Oct 7;20(10):e0014785. doi: 10.1371/journal.pntd.0014785 (PMC13674859; doi:10.1371/journal.pntd.0014785)
Supplement: Peer review history

# Peer review: Decision Letter 0

**DOI:** 10.1371/journal.pntd.0014785.r001

**Authors:** Michael Holbrook, David Simons

30 Jun 2026

PNTD-D-26-00967

Paediatric mpox during Sierra Leone's 2024 to 2025 clade IIb outbreak: a national surveillance study of 276 children

PLOS Neglected Tropical Diseases

Dear Dr. Ikoona,

Thank you for submitting your manuscript to PLOS Neglected Tropical Diseases. After careful consideration, we feel that it has merit but does not fully meet PLOS Neglected Tropical Diseases's publication criteria as it currently stands. Therefore, we invite you to submit a revised version of the manuscript that addresses the points raised during the review process.

Please submit your revised manuscript within by Aug 29 2026 11:59PM. If you will need more time than this to complete your revisions, please reply to this message or contact the journal office at plosntds@plos.org. When you're ready to submit your revision, log on to https://www.editorialmanager.com/pntd/ and select the 'Submissions Needing Revision' folder to locate your manuscript file.

Please include the following items when submitting your revised manuscript:

* A letter that responds to each point raised by the editor and reviewer(s). You should upload this letter as a separate file labeled 'Response to Reviewers'. This file does not need to include responses to any formatting updates and technical items listed in the 'Journal Requirements' section below.

* A marked-up copy of your manuscript that highlights changes made to the original version. You should upload this as a separate file labeled 'Revised Manuscript with Track Changes'.

* An unmarked version of your revised paper without tracked changes. You should upload this as a separate file labeled 'Manuscript'.

If you would like to make changes to your financial disclosure, competing interests statement, or data availability statement, please make these updates within the submission form at the time of resubmission. Guidelines for resubmitting your figure files are available below the reviewer comments at the end of this letter.

As the corresponding author, your ORCID iD is verified in the submission system and will appear in the published article. PLOS supports the use of ORCID, and we encourage all coauthors to register for an ORCID iD and use it as well. Please encourage your coauthors to verify their ORCID iD within the submission system before final acceptance, as unverified ORCID iDs will not appear in the published article. Only the individual author can complete the verification step; PLOS staff cannot verify ORCID iDs on behalf of authors.

We look forward to receiving your revised manuscript.

Kind regards,

David Simons

Academic Editor

PLOS Neglected Tropical Diseases

Michael Holbrook

Section Editor

PLOS Neglected Tropical Diseases

Shaden Kamhawi

co-Editor-in-Chief

PLOS Neglected Tropical Diseases

orcid.org/0000-0003-4304-636XX

Paul Brindley

co-Editor-in-Chief

PLOS Neglected Tropical Diseases

orcid.org/0000-0003-1765-0002

**Additional Editor Comments (if provided):**

Thank you for your submission, which I am recommending for Major Revisions. Please see the comments from the two reviewers below. They both agree that this is an important study on a paediatric cohort.

The review panel highlights several critical epidemiological and clinical additions that are required to improve the manuscript.

While this dataset represents a valuable national descriptive cohort of paediatric mpox from West Africa, several central conclusions currently extend beyond what passive surveillance data can support. In addition to addressing the individual reviewer comments, you should address the following methodology, data integrity, and interpretative issues in your submission.

1. Data Integrity & Denominator Reconciliation: The official Ministry of Health confirmed outbreak total for this period stands at 5,442 cases, yet your baseline denominator shrinks to 4,140 before isolating the paediatric cases. A PRISMA-style inclusion/exclusion flowchart would be helpful. You must explicitly document where and why these ~1,300+ records were dropped (e.g., missing age variables, discarded suspect entries).

Ensure that all table denominators are mathematically consistent. For instance, in Table 1, clarify why the mortality metric reads 2/130 for an n=131 cohort, and why the type of care category drops to 124. Missing or unreported fields must be accounted for explicitly.

2. Methodological & Spatial Biases: Your central geographic argument hinges on an elevated paediatric over-representation ratio in rural districts (Koinadugu, Falaba, Karene) to infer preferential household transmission. This metric is a mathematical artifact of case composition. Because Freetown (Western Area Urban) experienced an adult-centric surge driven by distinct sexual transmission networks, the capital’s adult denominator was inflated. This mechanically depressed the paediatric proportion in the urban zone and artificially elevated it in rural districts where adult networks were less active. You must temper this interpretation and explicitly state that this ratio reflects adult case composition rather than elevated paediatric incidence or true relative risk.

The hospitalisation rate for a Clade IIb epidemic is high. Your discussion must address two factors: first, that national operational response policy during the outbreak frequently mandated hospital admission for strict infection control and isolation purposes rather than clinical severity; and second, that passive surveillance via DHIS2 inherently captures a health-seeking cohort of severe cases, underrepresenting mild community cases.

3. Diagnostic Robustness & Clinical Confounders: The analytic cohort merges PCR-confirmed, probable, and unconfirmed clinician-diagnosed cases. In West African settings, severe varicella (chickenpox) and atypical measles are likely indistinguishable clinically from mpox in children under five. You must provide a clear, age-stratified breakdown of case verification status (PCR vs. Probable vs. Clinician-confirmed). If the 0–4 age group is heavily reliant on clinical diagnosis alone, this potential varicella contamination must be addressed as a limitation.

HIV status is completely absent from your variable list. Given that uncontrolled HIV is a primary driver of severe Clade IIb disease, hospitalisation, and mortality, it is not possible to compare clinical trajectories or case fatality rates between adults and children without discussing this. If the DHIS2 framework failed to capture this, it must be highlighted as a limitation.

4. Epidemiological Interpretations & Operational Reality: Because your study lacks direct contact tracing or secondary attack rate data, your assertion that this data proves household transmission must be softened to a hypothesis. There remain highly plausible alternative hyotheses, for example the transmission pathway in remote, heavily forested northern districts like Koinadugu and Falaba may be drive by zoonotic spillover from children interacting with or preparing wild rodent and bushmeat reservoirs.

You report a 9.4% paediatric vaccination rate and interpret this as an access gap. This overlooks national operational context, the deployed MVA-BN vaccine was restricted to individuals aged 12 and older. Most of your cohort was therefore ineligible. You must define what this surveillance tick-box actually captured, if there is a high likelihood that during data entry recorders checked this box for routine childhood immunisations (measles/polio), this variable must be cleaned or removed.

The striking age gradient in female vaginal lesions, rising from 7.4% in toddlers to nearly 40% in early adolescents (10–14 years) would benefit from clinical and contextual discussion. Clarify if exposure routes or safeguarding safety audits (child protection referrals, social work or forensic assessments) were captured by the surveillance architecture. If these institutional safety data are unavailable, this should be explicitly reported as an urgent limitation.

Finally, please ensure your revised manuscript treats these findings strictly as a valuable descriptive epidemiological slice of the outbreak. The language in your Author Summary and Conclusions must be adjusted to match the structural constraints and underlying uncertainties of passive surveillance data.

**Journal Requirements:**

If the reviewer comments include a recommendation to cite specific previously published works, please review and evaluate these publications to determine whether they are relevant and should be cited. There is no requirement to cite these works unless the editor has indicated otherwise.

1) Please upload all main figures as separate Figure files in .tif or .eps format. For more information about how to convert and format your figure files please see our guidelines:

https://journals.plos.org/plosntds/s/figures

2) In the online submission form, you indicated that All aggregate data needed to reproduce the reported tables are included in the manuscript. The underlying individual-level surveillance records cannot be shared publicly because they contain sensitive public health data. The records are held by the National Public Health Agency of Sierra Leone. Data access requests are subject to national data governance approval. Requests may be directed to the National Public Health Agency, Freetown, Sierra Leone, through the corresponding author.. All PLOS journals now require all data underlying the findings described in their manuscript to be freely available to other researchers, either

1. In a public repository

2. Within the manuscript itself

3. Uploaded as supplementary information.

This policy applies to all data except where public deposition would breach compliance with the protocol approved by your research ethics board. If your data cannot be made publicly available for ethical or legal reasons (e.g., public availability would compromise patient privacy), please explain your reasons by return email and your exemption request will be escalated to the editor for approval. Your exemption request will be handled independently and will not hold up the peer review process, but will need to be resolved should your manuscript be accepted for publication. One of the Editorial team will then be in touch if there are any issues.

3) We have amended your Competing Interest statement to comply with journal style. We kindly ask that you double check the statement and let us know if anything is incorrect.

**Reviewers' Comments:**

Reviewer's Responses to Questions

**Key Review Criteria Required for Acceptance?**

As you describe the new analyses required for acceptance, please consider the following:

**Methods**

-Are the objectives of the study clearly articulated with a clear testable hypothesis stated?

-Is the study design appropriate to address the stated objectives?

-Is the population clearly described and appropriate for the hypothesis being tested?

-Is the sample size sufficient to ensure adequate power to address the hypothesis being tested?

-Were correct statistical analysis used to support conclusions?

-Are there concerns about ethical or regulatory requirements being met?

Reviewer #1: (No Response)

Reviewer #2: full review posted under summary and general comments

**********

**Results**

-Does the analysis presented match the analysis plan?

-Are the results clearly and completely presented?

-Are the figures (Tables, Images) of sufficient quality for clarity?

Reviewer #1: (No Response)

Reviewer #2: Full review posted under summary and general comments

**********

**Conclusions**

-Are the conclusions supported by the data presented?

-Are the limitations of analysis clearly described?

-Do the authors discuss how these data can be helpful to advance our understanding of the topic under study?

-Is public health relevance addressed?

Reviewer #1: (No Response)

Reviewer #2: Full review posted under summary and general comments

**********

**Editorial and Data Presentation Modifications?**

Use this section for editorial suggestions as well as relatively minor modifications of existing data that would enhance clarity. If the only modifications needed are minor and/or editorial, you may wish to recommend “Minor Revision” or “Accept”.

Reviewer #1: (No Response)

Reviewer #2: Full review posted under summary and general comments

**********

**Summary and General Comments**

Use this section to provide overall comments, discuss strengths/weaknesses of the study, novelty, significance, general execution and scholarship. You may also include additional comments for the author, including concerns about dual publication, research ethics, or publication ethics. If requesting major revision, please articulate the new experiments that are needed.

Reviewer #1: (No Response)

Reviewer #2: This retrospective national surveillance analysis describes 276 children with mpox during Sierra Leone's 2024–2025 clade IIb G.1 outbreak. Contemporary paediatric data from African clade IIb epidemics remain limited, making this one of the larger national cohorts reported from West Africa. The manuscript addresses an important evidence gap in a literature that has largely been informed by historical clade I outbreaks and the predominantly adult-focused 2022 multicountry epidemic. The descriptive epidemiology is well presented, and the authors are appropriately transparent about several important limitations of the surveillance dataset. However, in its current form, several of the central conclusions extend beyond what the available data can support. In particular, the interpretation of the geographic analysis, the framing of vaccination findings, and the discussion of genital lesions require substantial revision.

Major comments

1. The geographic over-representation ratio does not support the conclusions drawn regarding household transmission. The central interpretation of the manuscript rests heavily on the paediatric over-representation ratio, defined as each district's share of paediatric cases divided by its share of all eligible cases. This is fundamentally a compositional metric. Because western area urban experienced a predominance of adult cases likely driven by sexual transmission networks, the denominator in that district is disproportionately inflated. Consequently, the paediatric proportion is mechanically reduced in western area urban and relatively increased in districts where adult sexual-network transmission was less prominent. The elevated ratios observed in Koinadugu, Falaba, and Karene therefore do not necessarily indicate increased paediatric risk or preferential household transmission. Rather, they may simply reflect differences in the underlying composition of adult cases across districts. Without district-level paediatric population denominators or measures of testing intensity, the metric cannot be interpreted as reflecting incidence or relative risk, a limitation the authors acknowledge but then largely set aside throughout the Results and Discussion.

I recommend that the authors: 1. explicitly state that this metric is expected to increase in districts where adult sexual-network transmission is comparatively less common;2.) substantially temper the discussion linking these findings to household transmission; and 3). where possible, incorporate district-level child population denominators or testing data to provide a more informative sensitivity analysis. At present, the data support describing geographic clustering but do not provide convincing evidence that household transmission was the dominant driver.

2. The interpretation of the vaccination findings does not adequately consider vaccine eligibility and supply constraints. The manuscript highlights low paediatric vaccination rates (12/128 children with known vaccination status) and interprets this primarily as a gap in access that should be addressed through expanded post-exposure vaccination. This interpretation overlooks a key contextual issue. The vaccine deployed during the Sierra Leone response was MVA-BN. National rollout occurred under severe supply constraints, with vaccination targeted primarily to frontline healthcare workers and selected high-risk populations. In addition, routine use was restricted to individuals aged 12 years and older. Nearly half of the paediatric cohort was younger than five years, and most children were below the age of eligibility. Accordingly, the observed differences between children and adults are likely explained, at least in part, by vaccine eligibility and limited vaccine availability rather than differential access alone.

The manuscript would be substantially strengthened by explicitly naming the vaccine used; describing the national eligibility criteria and supply limitations; restricting child-adult comparisons to age-eligible children where feasible, or clearly acknowledging that pooled analyses conflate eligibility with access; and reframing the discussion toward the more relevant policy question of off-label use in younger children during outbreaks, consistent with the WHO 2024 position paper already cited. Without this context, readers may incorrectly conclude that children simply failed to receive vaccines despite being eligible.

3. The age gradient in genital lesions is potentially important but receives insufficient discussion. One of the more striking observations in the manuscript is the progressive increase in documented vaginal lesions across age groups:

• 7.4% among girls aged 0–4 years

• 24.2% among girls aged 5–9 years

• 38.7% among girls aged 10–14 years

The manuscript reports the pooled proportion (20.3%) and briefly notes that genital lesions do not establish sexual transmission before moving on. Given the epidemiology of clade IIb outbreaks, this deserves considerably more discussion. While genital lesions in younger children may plausibly reflect autoinoculation or disseminated disease, the same explanation becomes less straightforward among early adolescents. The increasing prevalence with age raises important clinical and safeguarding considerations that cannot simply be acknowledged in passing. The authors should clarify whether exposure history or suspected route of acquisition was recorded; whether any safeguarding evaluations, child-protection referrals, forensic examinations, or social-work assessments were performed; whether these data are unavailable within the surveillance system; and whether results for the 10–14-year age group should be presented separately rather than being incorporated into a broad paediatric category. If no safeguarding data were collected, this should be acknowledged explicitly as an important limitation.

4. The clinical description remains relatively superficial. The manuscript's greatest potential contribution lies in its national paediatric cohort, yet the clinical description is limited. Apart from secondary bacterial skin infection, one case of ARDS, and one case of severe dehydration, relatively little clinical information is presented. The surveillance system may not capture all clinically relevant variables, but the manuscript should clarify exactly which clinical fields were available within DHIS2 and whether all available information has been extracted. If feasible, targeted chart abstraction for hospitalized children or the fatal cases would substantially increase the manuscript's clinical value by providing information on lesion distribution, mucosal or ocular involvement, nutritional status, treatment received, disease severity, and clinical course.

5. The mortality findings would benefit from additional clinical context. Three paediatric deaths occurred during the outbreak. While the authors appropriately report the case fatality rate, the estimate is necessarily imprecise given the small number of events. Rather than emphasizing comparisons with adults, the manuscript would be considerably strengthened by providing brief descriptions of each fatal case, including age, sex, district, confirmation status, relevant comorbidities or nutritional status (where available), complications, treatment setting, and immediate cause of death. If these data cannot be retrieved, the mortality discussion should be correspondingly more cautious.

6. Laboratory confirmation should be reported by age group. The analytic cohort combines PCR-confirmed, probable, and clinician-confirmed cases. However, the distribution of these categories within the paediatric cohort is not presented. This is important because vesiculopustular illnesses such as varicella, impetigo, hand-foot-and-mouth disease, and scabies remain important differential diagnoses in young children. Clinician-confirmed cases without laboratory confirmation are therefore particularly susceptible to misclassification. The authors should report confirmation status by paediatric age group. If a substantial proportion of younger children were classified solely on clinical grounds, this should be discussed as a limitation when interpreting the predominance of children under five years and the geographic distribution.

Minor comments

• Hospitalization likely reflects isolation policy as well as disease severity. Throughout many African mpox responses, hospital admission has frequently served infection-control purposes in addition to clinical management. The higher hospitalization rate observed among children may therefore reflect operational policy rather than greater disease severity. This distinction should be acknowledged.

• The comparison with the reported 1.3% paediatric proportion from the multicountry outbreak should acknowledge differences in both age definitions and surveillance strategies, not age cutoffs alone.

• The child-adult comparisons are explicitly exploratory but are presented primarily through p-values. Consider emphasizing effect estimates and confidence intervals and acknowledging that no adjustment for multiple comparisons was performed.

• Data completeness differs substantially across variables, particularly for vaccination and care status. Please report completeness separately for children and adults and discuss the potential direction of resulting bias.

• The Methods describe comparing onset-to-care intervals using the Wilcoxon rank-sum test, but these results are not presented. Either include the analysis or remove it from the Methods.

• The interpretation accompanying Figure 2 should be revised to align with a more cautious discussion of the over-representation metric.

• Finally, the language used in the author Summary and conclusions should mirror the uncertainty acknowledged elsewhere in the manuscript. Household exposure is certainly plausible and likely contributed substantially to paediatric transmission, but the available surveillance data do not allow this pathway to be demonstrated directly.

**********

PLOS authors have the option to publish the peer review history of their article (what does this mean?). If published, this will include your full peer review and any attached files.

If you choose “no”, your identity will remain anonymous but your review may still be made public.

**Do you want your identity to be public for this peer review?**  For information about this choice, including consent withdrawal, please see our Privacy Policy.

Reviewer #1: No

Reviewer #2: No

[NOTE: If reviewer comments were submitted as an attachment file, they will be attached to this email and accessible via the submission site. Please log into your account, locate the manuscript record, and check for the action link "View Attachments". If this link does not appear, there are no attachment files.]

**Figure resubmission:**

**Reproducibility:**

To enhance the reproducibility of your results, we recommend that authors of applicable studies deposit laboratory protocols in protocols.io, where a protocol can be assigned its own identifier (DOI) such that it can be cited independently in the future. Additionally, PLOS ONE offers an option to publish peer-reviewed clinical study protocols. Read more information on sharing protocols at https://plos.org/protocols?utm_medium=editorial-email&utm_source=authorletters&utm_campaign=protocols

Attachment Submitted filename: Reviewer recommendations_ped_mpox_SL_2026_.docx

# Author comment: Author response to Decision Letter 1

**DOI:** 10.1371/journal.pntd.0014785.r002

16 Jul 2026

Attachment Submitted filename: Response_to_Reviewers.docx

# Peer review: Decision Letter 1

**DOI:** 10.1371/journal.pntd.0014785.r003

**Authors:** Michael Holbrook, David Simons

9 Sep 2026

Response to Reviewers Revised Manuscript with Track Changes Manuscript'.

Shaden Kamhawi

co-Editor-in-Chief

PLOS Neglected Tropical Diseases

orcid.org/0000-0003-4304-636XX

Paul Brindley

co-Editor-in-Chief

PLOS Neglected Tropical Diseases

orcid.org/0000-0003-1765-0002

**Journal Requirements:**

1) In the online submission form, you indicated that All aggregate data needed to reproduce the reported tables are included in the manuscript. The underlying individual-level surveillance records cannot be shared publicly because they contain sensitive public health data. The records are held by the National Public Health Agency of Sierra Leone. Data access requests are subject to national data governance approval. Requests may be directed to the National Public Health Agency, Freetown, Sierra Leone, through the corresponding author.. All PLOS journals now require all data underlying the findings described in their manuscript to be freely available to other researchers, either

1. In a public repository

2. Within the manuscript itself

3. Uploaded as supplementary information.

This policy applies to all data except where public deposition would breach compliance with the protocol approved by your research ethics board. If your data cannot be made publicly available for ethical or legal reasons (e.g., public availability would compromise patient privacy), please explain your reasons by return email and your exemption request will be escalated to the editor for approval. Your exemption request will be handled independently and will not hold up the peer review process, but will need to be resolved should your manuscript be accepted for publication. One of the Editorial team will then be in touch if there are any issues.

**Reviewers' comments:**

**Key Review Criteria Required for Acceptance?**

As you describe the new analyses required for acceptance, please consider the following:

**Methods**

-Are the objectives of the study clearly articulated with a clear testable hypothesis stated?

-Is the study design appropriate to address the stated objectives?

-Is the population clearly described and appropriate for the hypothesis being tested?

-Is the sample size sufficient to ensure adequate power to address the hypothesis being tested?

-Were correct statistical analysis used to support conclusions?

-Are there concerns about ethical or regulatory requirements being met?

Reviewer #2: (No Response)

**********

**Results**

-Does the analysis presented match the analysis plan?

-Are the results clearly and completely presented?

-Are the figures (Tables, Images) of sufficient quality for clarity?

Reviewer #2: (No Response)

**********

**Conclusions**

-Are the conclusions supported by the data presented?

-Are the limitations of analysis clearly described?

-Do the authors discuss how these data can be helpful to advance our understanding of the topic under study?

-Is public health relevance addressed?

Reviewer #2: (No Response)

**********

**Editorial and Data Presentation Modifications?**

Use this section for editorial suggestions as well as relatively minor modifications of existing data that would enhance clarity. If the only modifications needed are minor and/or editorial, you may wish to recommend “Minor Revision” or “Accept”.

Reviewer #2: (No Response)

**********

**Summary and General Comments**

Use this section to provide overall comments, discuss strengths/weaknesses of the study, novelty, significance, general execution and scholarship. You may also include additional comments for the author, including concerns about dual publication, research ethics, or publication ethics. If requesting major revision, please articulate the new experiments that are needed.

Reviewer #2: Summary

The authors have substantially reworked this manuscript rather than making incremental edits. I have one substantive point remaining on the vaccination discussion, and one structural point related to the cohort change that I'd like the authors to confirm does not reopen any of the geographic or diagnostic concerns from my original review in a new form.

Major comment

Vaccination discussion still stops short of the off-label-use framing requested. The authors have made the factual corrections I asked for: MVA-BN is named, the targeted single-dose campaign restricted to selected groups aged 12 years or older is described, and the paediatric vaccination analysis is now restricted to age-eligible children (12–14 years), with the pooled-access interpretation removed. This resolves the main statistical concern. However, my original comment also asked the authors to reframe the discussion toward the more relevant policy question off-label or expanded-eligibility use of MVA-BN in younger children during outbreaks, consistent with the WHO 2024 position paper already cited in the manuscript (ref 18). As written, the Discussion paragraph on vaccination ends at the observation that the data cannot support coverage, access, or effectiveness estimates. It does not engage with the policy question of whether and how younger, currently ineligible children (nearly half the paediatric cohort was under 5) should be considered for vaccination in future outbreaks, despite WHO's position paper being available to ground that discussion. Given that the manuscript's stated purpose includes informing outbreak response, I'd ask the authors to add 2–3 sentences engaging directly with this question, even if only to note the evidence gap and the need for policy guidance on off-label paediatric use during active transmission.

Minor comment - Please confirm the cohort revision does not reopen prior concerns in new form. The analytic cohort changed from 276 to 415 children between submissions, in response to denominator-reconciliation comments. The explanation provided (rebuilt from the populated case-classification field; S1 Fig traces record flow; no eligible records excluded) is transparent and reasonable, and I have no concerns about the reconstruction itself. However, since my original review was conducted against the 276-child cohort, none of the original tables map directly onto the revised ones. I'd ask the authors to confirm in their response that: the district-level notification patterns (Western Area Urban, Koinadugu, Falaba) are consistent in direction and magnitude between the two cohort versions, and

he age-specific laboratory-confirmation proportions in the new Table 1 do not reveal a different pattern of clinical-only diagnosis in young children than was apparent in the original 276-case cohort. This is a confirmatory request rather than a sign of a new problem; I raise it only because the underlying data extract changed substantially and I want to be sure nothing shifted qualitatively.

I thank the authors for a careful and responsive revision.

**********

PLOS authors have the option to publish the peer review history of their article (what does this mean?). If published, this will include your full peer review and any attached files.

If you choose “no”, your identity will remain anonymous but your review may still be made public.

**Do you want your identity to be public for this peer review?**  For information about this choice, including consent withdrawal, please see our Privacy Policy.

Reviewer #2: No

**Figure resubmission:**

**Reproducibility:** -->To enhance the reproducibility of your results, we recommend that authors of applicable studies deposit laboratory protocols in protocols.io, where a protocol can be assigned its own identifier (DOI) such that it can be cited independently in the future. Additionally, PLOS ONE offers an option to publish peer-reviewed clinical study protocols. Read more information on sharing protocols at https://plos.org/protocols?utm_medium=editorial-email&utm_source=authorletters&utm_campaign=protocols-->?>

# Author comment: Author response to Decision Letter 2

**DOI:** 10.1371/journal.pntd.0014785.r004

10 Sep 2026

Attachment Submitted filename: Response_to_Reviewers_auresp_2.docx

# Editor report: Decision Letter 2

**DOI:** 10.1371/journal.pntd.0014785.r005

**Authors:** Michael Holbrook, David Simons

27 Sep 2026

Dear Dr Ikoona,

We are pleased to inform you that your manuscript 'Paediatric mpox during Sierra Leone's 2024 to 2025 clade IIb outbreak: a national surveillance and outbreak-investigation study of 415 children' has been provisionally accepted for publication in PLOS Neglected Tropical Diseases.

Before your manuscript can be formally accepted you will need to complete some formatting changes, which you will receive in a follow up email. A member of our team will be in touch with a set of requests.

Please note that your manuscript will not be scheduled for publication until you have made the required changes, so a swift response is appreciated.

IMPORTANT: The editorial review process is now complete. PLOS will only permit corrections to spelling, formatting or significant scientific errors from this point onwards. Requests for major changes, or any which affect the scientific understanding of your work, will cause delays to the publication date of your manuscript.

Should you, your institution's press office or the journal office choose to press release your paper, you will automatically be opted out of early publication. We ask that you notify us now if you or your institution is planning to press release the article. All press must be co-ordinated with PLOS.

Thank you again for supporting Open Access publishing; we are looking forward to publishing your work in PLOS Neglected Tropical Diseases.

Best regards,

David Simons

Academic Editor

PLOS Neglected Tropical Diseases

Michael Holbrook

Section Editor

PLOS Neglected Tropical Diseases

Shaden Kamhawi

co-Editor-in-Chief

PLOS Neglected Tropical Diseases

orcid.org/0000-0003-4304-636XX

Paul Brindley

co-Editor-in-Chief

PLOS Neglected Tropical Diseases

orcid.org/0000-0003-1765-0002

***********************************************************

Thank you for your and our reviewers engagement with the peer-review process I am happy to recommend progression to acceptance of this work.

p.p1 {margin: 0.0px 0.0px 0.0px 0.0px; line-height: 16.0px; font: 14.0px Arial; color: #323333; -webkit-text-stroke: #323333}span.s1 {font-kerning: none

# Editor report: Acceptance letter

**DOI:** 10.1371/journal.pntd.0014785.r006

**Authors:** Michael Holbrook, David Simons

Dear Dr Ikoona,

We are delighted to inform you that your manuscript, "Paediatric mpox during Sierra Leone's 2024 to 2025 clade IIb outbreak: a national surveillance and outbreak-investigation study of 415 children," has been formally accepted for publication in PLOS Neglected Tropical Diseases.

We have now passed your article onto the PLOS Production Department who will complete the rest of the publication process. All authors will receive a confirmation email upon publication.

The corresponding author will soon be receiving a typeset proof for review, to ensure errors have not been introduced during production. Please review the PDF proof of your manuscript carefully, as this is the last chance to correct any scientific or type-setting errors. Please note that major changes, or those which affect the scientific understanding of the work, will likely cause delays to the publication date of your manuscript. Note: Proofs for Front Matter articles (Editorial, Viewpoint, Symposium, Review, etc...) are generated on a different schedule and may not be made available as quickly.

Soon after your final files are uploaded, the early version of your manuscript will be published online unless you opted out of this process. The date of the early version will be your article's publication date. The final article will be published to the same URL, and all versions of the paper will be accessible to readers.

For Research Articles, you will receive an invoice from PLOS for your publication fee after your manuscript has reached the completed accept phase. If you receive an email requesting payment before acceptance or for any other service, this may be a phishing scheme. Learn how to identify phishing emails and protect your accounts at https://explore.plos.org/phishing.

Thank you again for supporting open-access publishing; we are looking forward to publishing your work in PLOS Neglected Tropical Diseases.

Best regards,

Shaden Kamhawi

co-Editor-in-Chief

PLOS Neglected Tropical Diseases

Paul Brindley

co-Editor-in-Chief

PLOS Neglected Tropical Diseases
